# Mutacin H-29B is identical to mutacin II (J-T8)

**DOI:** 10.1186/1471-2180-6-36

**Published:** 2006-04-20

**Authors:** Guillaume Nicolas, Hélène Morency, Gisèle LaPointe, Marc C Lavoie

**Affiliations:** 1 Département de Biochimie et Microbiologie, Faculté des Sciences et Génie, Université Laval, Quebec, Quebec, G1K 7P4, Canada; 2 Centre de Recherche en Sciences et Technologie du Lait (STELA), Institut des Nutraceutiques et des Aliments Fonctionnels (INAF), Faculté des Sciences de 1' Agriculture et de 1' Alimentation, Université Laval, Québec, G1K 7P4, Canada; 3 Department of Biological and Chemical Sciences, Faculty of Pure and Applied Sciences, The University of the West Indies, Cave Hill Campus, Bridgetown, BB 11000, Barbados

## Abstract

**Background:**

*Streptococcus mutans *produces bacteriocins named mutacins. Studies of mutacins have always been hampered by the difficulties in obtaining active liquid preparations of these substances. Some of them were found to be lantibiotics, defined as bacterial ribosomally synthesised lanthionine-containing peptides with antimicrobial activity. The goal of this study was to produce and characterize a new mutacin from *S. mutans *strain 29B, as it shows a promising activity spectrum against current human pathogens.

**Results:**

Mutacin H-29B, produced by *S. mutans *strain 29B, was purified by successive hydrophobic chromatography from a liquid preparation consisting of cheese whey permeate (6% w/v) supplemented with yeast extract (2%) and CaCO_3 _(1%). Edman degradation revealed 24 amino acids identical to those of mutacin II (also known as J-T8). The molecular mass of the purified peptide was evaluated at 3246.08 ± 0.1 Da by MALDI-TOF MS.

**Conclusion:**

A simple procedure for production and purification of mutacins along with its characterization is presented. Our results show that the amino acid sequence of mutacin H-29B is identical to the already known mutacin II (J-T8) over the first 24 residues. *S. mutans *strains of widely different origins may thus produce very similar bacteriocins.

## Background

Bacteriocins are ribosomally synthesised proteinaceous bactericidal substances produced by bacteria [1]. Mutacins are bacteriocins produced by *Streptococcus mutans *[2] that are active against Gram-positive and some Gram-negative bacteria [3,4]. To date, four types of mutacin have been described: the mono- or di-peptide lantibiotic and the mono- or di-peptide non-lantibiotic mutacin [5-8]. Lantibiotics contain unusual amino acids such as Lan, BM-Lan and dehydrated amino acid residues [9]. Posttranslational modification of the prepeptide occurs through amino acid dehydration, formation of thioether bridges and cleavage of the leader peptide during transport outside of the cell [9].

We previously classified mutacin-producing strains into 24 groups (designated A to X) and 7 clusters according to their activity spectra against bacterial pathogens and their immunity towards other mutacins [10,4]. The strain 29B producing mutacin H-29B was found to be closely related in its activity spectrum with strain T8 which produces mutacin II (also known as J-T8) [4]. The DNA probe designed to hybridize with the *mut*A gene of mutacin II (J-T8) also hybridised with the DNA of strain 29B [11]. We present here a simple procedure for production and purification of mutacin H-29B, along with its partial characterization. The results show that the amino acid sequence of mutacin H-29B is identical to that of mutacin II (J-T8) over the first 24 residues. *S. mutans *strains of widely different origins may thus produce similar bacteriocins.

## Results and discussion

### Thermostability and pH stability

The activity of crude preparations of mutacin H-29B, calculated at 6400 AU/ml (Table 1) at pH 4, was not reduced in boiling water (100°C) after 1 h or after autoclaving (121°C, 15 min), but lost 20% of its activity after 2 h at 100°C. All mutacin activity was retained at pH 2–4 after 24 h, but was reduced to 80% at pH 5–7, to 60% at pH 8–9, and to 30% at pH 9–12. These results are in agreement with previously published results obtained on the activity of the mutacin-producing strain *S. mutans *29B [10].

### Purification and characterization of the mutacin H-29B

Purification of mutacin H-29B was achieved by means of three hydrophobic chromatography steps (Table 1). The final specific activity of the purified mutacin H-29B was 6.4 × 10^6 ^AU/mg. As for mutacin II (J-T8) [12,13], the amino acid sequence of the purified mutacin H-29B was blocked at position 9 without alkaline ethanethiol treatment, indicating the presence of modified residues and the lantibiotic nature of mutacin H-29B. This N-terminal sequence was found to be identical to mutacin II (J-T8) [12]. Balakrishnan et al. [14] also reported that mutacin M 19 presents the same N-terminal amino acid sequence. The absence of methionine at the N-terminus suggests that mutacin H-29B may be processed from a precursor form, as reported for lantibiotics in general [9].

Edman degradation performed after alkaline ethanethiol derivatization [15] revealed a total of 24 amino acids. The complete sequence of mutacin H-29B confirms its identity with the mutacin II (J-T8). Modified amino acids were obtained in positions 10, 12, 15, and 19. A BMSEC signal seen as a doublet signal in cycle 10 indicates the presence of an Abu or a dhB [15]. A SEC signal was observed in positions 12, 15, and 19 during sequencing, which indicate the presence of a dhA residue [15]. In each case, the SEC signal was not accompanied by a DSER signal. At this stage, we can not tell if the dhA or Abu residues are implicated or not in the formation of thioether bridges. A deamidation was also observed for the Asn and Gln in positions 1, 5 and 18, which can result from the treatment with alkaline ethanethiol. A Phe residue was observed as a minor amino acid in position 25, probably resulting from the carryover effect of the sequencing procedure.

**Figure 1:**
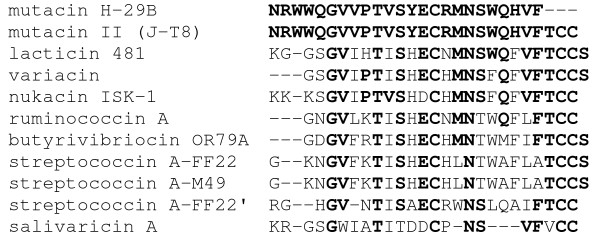
Multiple sequence alignment of mutacin H-29B with homologous type AII lantibiotics. Only propeptides are shown. Conserved amino acids are in bold-type. Bacteriocin ruminococcin A is produced by *Ruminococcus gnavus *[25], butyrivibriocin OR79A by *Butyrivibrio fibrisolvens *[26], salivaricin A by *Streptococcus salivarius *[27], lacticin 481 by *Lactococcus lactis *[28], variacin by *Micrococcus varians *[29], nukacin ISK-1 by *Staphylococcus warneri *[30], mutacin II (J-T8) [16], and streptococcin A-M49, A-FF22 and A-FF22' are produced by *Streptococcus pyogenes *[31, 32].

Comparison of the mutacin H-29B sequence with known bacteriocins revealed the high levels of homology to group AII lantibiotics (Figure 1). However, the TCCS or TCC motif present at the C-terminus of other AII lantibiotics was not detected by Edman degradation for mutacin H-29B. The DNA sequence of the gene coding for mutacin H-29B will confirm whether the TCC motif is also present at the C-terminus of mutacin H-29B.

The molecular mass of mutacin H-29B determined by MALDI-TOF MS (3246.08 ± 0.1 Da) is closely related to the one estimated for mutacin II (J-T8) (3244.64 ± 1.15 Da) [13,16]. This strongly suggested that these two mutacins are identical, including the three terminal amino acids. Thus, the experimentally-demonstrated thioether bridge pattern of mutacin II (J-T8) [16] can be proposed for mutacin H-29B. These would consist of one BM-Lan bridge between the residues in positions 10 (Thr) and 15 (Cys), one Lan bridge between residues 12 (Ser) and 26 (Cys) as well as a second Lan bridge between residues 19 (Ser) and 27 (Cys).

Different strains can produce the same mutacin: *S. mutans *strains T8, isolated in Australia; UA96, isolated in Alabama, USA; and 29B, isolated in Quebec, Canada [10,17], produce mutacin II (J-T8) ([12,14], this paper); *S. mutans *strains CH43 and UA140 produce mutacin I [7,18]; and *S. mutans *JH 1140, isolated in Florida USA [19], and UA787, isolated in Alabama, USA [20], produce the same mutacin, that only differs from mutacin B-Ny266 by only two amino acids [5].

Genes coding for mutacin biosynthesis have all been located on the chromosome so far [7,8,18-22]. The mutacin II (J-T8) gene cluster has been identified and successfully transferred to a non-producing strain by transformation [22]. Evolution of the mutacin-lantibiotics from a common ancestor and its propagation via mobile genetic elements is suggested by further observations. A silent transposase gene was reported in the upstream region of the genes coding for mutacin II (J-T8) production [22]. Yonezawa and Kuramitsu (2005) [8] also observed direct repeats sequences flanking the *Smb *locus with similarity to those of transposase genes directly downstream of the locus. Sequences flanking the *Smb *locus were found adjacent in the UA159 *S. mutans *genome (GenBank Accession No AE014133). Mutacin-lantibiotic coding genes could perhaps be transferred via mobile genetic elements.

## Conclusion

Mutacin (H-29B) was isolated from an active liquid culture of *S. mutans *strain isolated in Quebec (29B) and proved to be identical to a previously characterized mutacin II (alias J-T8). Mutacin-lantibiotic production by *S. mutans *seems to be widespread despite the fact that the reference genome strain for *S. mutans *(UA159) does not seem to produce any of the mutacin lantibiotics characterized to date.

## Methods

### Bacterial strains and media

*Streptococcus mutans *29B was previously described in Parrot et al. [17] and *Micrococcus luteus *ATCC 272 was from the American Type Culture Collection (Manassas, VA, USA). They were routinely grown aerobically at 37°C in TSB (Difco laboratories, Detroit, MI, USA) supplemented with 0.3% of Yeast Extract (Becton Dickinson & Co., Cockeysville, MD) (TSBYE) or on TSA (Difco) plates enriched with 0.3% yeast extract (TSAYE).

### Mutacin production

An overnight preculture in TSBYE was used to inoculate (1%) 2 L of medium consisting of cheese whey permeate 6% (w/v) (kind gift from Agropur agroalimentary Coop, Granby, QC, Canada) supplemented with 1% CaCO_3 _(Anachemia, Montreal, QC, Canada) and 2% yeast extract (Institut Rosell Montreal, QC, Canada) (SWP). The culture was incubated 48 h at 37°C under aerobic conditions [23].

### Determination of inhibitory activity

Mutacin activity was determined as describe previously [5] with the following modification: 2-fold dilutions were prepared in acidified (pH 2) peptone water (0.5%) [23]. The specific activity of each preparation was expressed in AU/mg protein. Protein concentration was determined using the BioRad DC protein assay (BioRad, Mississauga, ON, Canada).

### Thermostability and pH stability

Crude mutacin (supernatant of SWP medium) was submitted to different pH conditions (pH 2 to pH 11) adjusted with 4 N HC1 or 4 N NaOH. After 2 h and 24 h at room temperature, residual mutacin activity was assayed by the spot test described above. Non-inoculated sterile SWP medium adjusted in the same conditions of the tested sample was used as negative control. Thermostability was assayed by determining the residual activity of crude mutacin samples adjusted at pH 4 and placed in boiling water for up to 120 min and after autoclaving.

### Mutacin H-29B extraction and purification

After 48 h of incubation at 37°C, the pH of the culture was adjusted to 2.0 with 4 N HC1 and incubated for 2 h at room temperature to desorb the mutacin [23]. The culture was then centrifuged at 10,000 × *g *for 10 min and heated at 70°C for 20 min to destroy the remaining cells and enzyme activity. The purification steps were essentially those previously used for the purification of mutacin B-Ny266 [5] with the following modification: concentration was carried out by adsorption to XAD-7 Amberlite (Sigma Chemical CO., St Louis, MO, USA). Hydrophobic chromatography on Sep-Pak^® ^Vac 35 cc (10 g) t-C_18 _Cartridges (Waters Corporation, Milford, MA, USA) was performed with the pooled active fractions diluted to 35% in MeOH in 10 mM HCl. Active fractions were recovered with an elution gradient of 50%–60% MeOH in 10 mM HC1. The final purification step was carried out by reverse phase chromatography (RP)-HPLC (Beckman Gold Model, Coulter Canada Inc., Missisauga, ON, Canada) using an analytical C_18 _column (Luna 5 μ C18(2), 250 × 4.6 mm, 4 × 3.0 mm, Phenomenex, Torrance, CA, USA). Elution was carried out with 5% acetonitrile in 0.1% TFA and 60% acetonitrile in 0.1% TFA and recorded at 214 nm.

Several re-injections of the same sample were made until a unique peak was obtained. Active fractions were dried in a Speed-Vac^® ^concentrator (Model SC110A, Savant Instrument Inc. Farmingdale, NY) and kept at -20°C for further analyses.

### Molecular mass determination

Molecular mass was determined for the purified HPLC fraction by MALDI-TOF MS on a Voyager DE-PRO (Applied Biosystems, Foster City, CA, USA) at the Peptide Synthesis & Protein Sequencing Core facility of Eastern Quebec, QC, Canada.

### Sequence determination

Automated Edman degradation was performed on a protein sequencer (ABI Procise cLC, Applied Biosystems, Foster City, CA, USA) after alkaline ethanethiol derivatization [15]. Pure mutacin B-Ny266 was used as control [5]. Homology searches were carried out with the National Center of Biotechnology Information (rps-BLAST search, with Expect value = 0.01 and Search mode = multiple hits, 1 pass) [24].

### Protein sequence accession number

The protein sequence data of mutacin H-29B appears in the Swiss-Prot and TrEMBL knowledgebase under the accession number P84110.

## Abbreviations

Abu, 2-aminobutyric acid; ATCC, American Type Culture Collection; AU, arbitrary units; BM-Lan, β-methyllanthionine; BMSEC, β-methyl-S-ethylcysteine; dhA, 2,3-didehydroalanine; dhB, 2,3-didehydrobutyrine; DSER, PTH-dithiothreitiol adduct of dhA; Lan, lanthionine; MALDI-TOF MS, Matrix Assisted Laser Desorption lonisation-Time of Flight Mass Spectroscopy; PTH, phenylthiohydantoin; RP-HPLC, reverse-phase high-pressure liquid chromatography; SEC, S-ethylcysteine; TFA, trifluoroacetic acid; TSAYE, trypticase soy agar yeast extract; TSBYE, trypticase soy broth yeast extract.

## Authors' contributions

Guillaume Nicolas participated in project conception, is the main manipulator of the experiments, evaluated MS and sequencing data and drafted the manuscript. Helene Morency participated in the purification of the peptide mutacin H-29B. Gisèle LaPointe designed and supervised the molecular analyses and participated in manuscript preparation. Marc C. Lavoie conceived the study and participated in its design and coordination as well as manuscript preparation. All authors read and approved the final manuscript.

## Figures and Tables

**Table 1:** Purification of mutacin H-29B by hydrophobic chromatography.

Step	Volume (mL)	Activity (AU/mL)	Total Protein (mg)	Total activity (AU. 10^3^)	Specific activity (AU/mg)	Yield (%)	Purification (fold)
Culture supernatant	1250	6400	10000	8000	800	100	1
XAD-7 Amberlite	300	6400	97.5	1920	20000	24	25
Sep-Pak C_18_	95	12800	1.9	1216	6.4 × 10^5^	15.2	800
C_18 _RP-HPLC	1	6.4 × 10^5^	0.1	640	6.4 × 10^6^	8	8000
